# Strength of baseline inter-trial correlations forecasts adaptive capacity in the vestibulo-ocular reflex

**DOI:** 10.1371/journal.pone.0174977

**Published:** 2017-04-05

**Authors:** Kara H. Beaton, Aaron L. Wong, Steven B. Lowen, Mark Shelhamer

**Affiliations:** 1 Department of Otolaryngology–Head and Neck Surgery, The Johns Hopkins University School of Medicine, Baltimore, MD, United States of America; 2 Department of Neurology, The Johns Hopkins University School of Medicine, Baltimore, MD, United States of America; 3 Department of Psychiatry, McLean Hospital, Harvard Medical School, Belmont, MA, United States of America; 4 Department of Biomedical Engineering, The Johns Hopkins University School of Medicine, Baltimore, MD, United States of America; University of Muenster, GERMANY

## Abstract

Individual differences in sensorimotor adaptability may permit customized training protocols for optimum learning. Here, we sought to forecast individual adaptive capabilities in the vestibulo-ocular reflex (VOR). Subjects performed 400 head-rotation steps (400 trials) during a baseline test, followed by 20 min of VOR gain adaptation. All subjects exhibited mean baseline VOR gain of approximately 1.0, variable from trial to trial, and showed desired reductions in gain following adaptation with variation in extent across individuals. The extent to which a given subject adapted was inversely proportional to a measure of the *strength and duration of baseline inter-trial correlations* (β). β is derived from the decay of the autocorrelation of the sequence of VOR gains, and describes how strongly correlated are past gain values; it thus indicates how much the VOR gain on any given trial is informed by performance on previous trials. To maximize the time that images are stabilized on the retina, the VOR should maintain a gain close to 1.0 that is adjusted predominantly according to the most recent error; hence, it is not surprising that individuals who exhibit smaller β (weaker inter-trial correlations) also exhibited the best adaptation. Our finding suggests that the temporal structure of baseline behavioral data contains important information that may aid in forecasting adaptive capacities. This has significant implications for the development of personalized physical therapy protocols for patients, and for other cases when it is necessary to adjust motor programs to maintain movement accuracy in response to pathological and environmental changes.

## Introduction

Meaningful interactions with the environment require integration of sensory inputs coupled with highly coordinated motor outputs. The inherent plasticity in the sensorimotor system provides remarkably adaptable motor control that facilitates compensation for movement errors that arise from both internal (e.g., pathological) and external (e.g., environmental) perturbations. With time, the body readily modifies behavior in response to extreme challenges, such as vestibular lesions or the changing gravity levels (g-levels) associated with space flight, as well as sporadic demands that occur during everyday life, such as donning new reading glasses or fatigue during exercise. Furthermore, even an accurate, highly functioning sensorimotor system continues to actively fine-tune motor responses to optimize performance [1]. Hence, the adaptive capabilities of the sensorimotor system enable high performance under a wide variety of circumstances.

While healthy persons generally show favorable adaptation when presented with a miscalibrated sensorimotor behavior, individual differences exist: some people adapt faster or more fully than others. Similarly, vestibular patients recovering from labyrinthectomies–while invoking a wide range of compensatory mechanisms instead of or in addition to recovery of the VOR *per se*–require varying amounts of rehabilitation therapy, and some take longer to recover than others [2, 3]. Astronauts enduring the same flight profiles respond differently to changes in g-level, and there is great variability in symptoms and functional impairments from one crewmember to the next [4, 5]. The ability to know *a priori* which vestibular patients might benefit from which gaze-stability paradigms, for instance, would enable more effective targeting of training and therapy and produce better outcomes. Therefore, the primary objective of this study was to search for baseline performance metrics that can be linked to adaptive capabilities.

Three recent studies have suggested that variability in baseline performance can be related to motor adaptation, reviewed subsequently. Variability in task performance is an inherent feature of all biological systems, manifest commonly as fluctuations in motor performance across repetitions of a task [6]. Traditionally, this “noise” has been deemed a random process, of little functional use [7, 8]. However, these recent investigations reveal that variability may represent a deliberate, actively regulated process that can facilitate both flexibility and adaptability [9, 10]. Although the definitions of variability described in these studies represent different system properties, the idea that a parameter that has been traditionally dismissed as inconvenient or even artifactual might forecast adaptive performance is both intriguing and unexpected.

One study, by Wu and colleagues [11], examined adaptation of arm-movement trajectories during a reaching task by means of reward-based learning. Subjects made repeated reaching movements between two targets without (at baseline) and with (during adaptive training) error feedback (reward). Performance was quantified by deviations between ideal and actual movement trajectories. The authors found that individuals with larger variability (standard deviation) in baseline performance exhibited faster learning during the training period. This finding can be interpreted by considering variability to be an exploratory process by which new motor patterns can be tested.

In contrast, Chaisanguanthum and colleagues [12] showed that variability in human pitching movements and monkey reaching movements can be decomposed into two parts, one that changes slowly and one that changes more rapidly on a trial-by-trial basis. They suggested that the dynamics of the slowly varying component are consistent with an underlying error-correction process that was identified in a prior study of reaching adaptation. In particular, these data imply that baseline variability and adaptation both arise from processes that exhibit a trade-off between stable (persistent) and flexible (variable) behavior to allow the system to maintain a sufficient level of performance while simultaneously searching for better options to avoid local minima [13, 14].

Finally, recent work in our laboratory has demonstrated a direct link between the inter-trial correlations observed in a baseline task and adaptive capabilities in saccades [15]. Inter-trial correlations depend on the temporal ordering of trials, and provide a measure of how strongly the performance of trials in the past influences the planning of the current trial. This measure of variability is distinct from that of gross system variability (i.e., dispersion about the central mean) as employed by Wu and colleagues. In fact, we found no relationship between gross variability (standard deviation) and adaptation. Instead, individuals who exhibited stronger long-term inter-trial correlations during our baseline saccade task adapted the fastest. This suggests that adaptation in this case depends on the ability to retain a longer history of error-correction information; better retention (larger correlations) leads to more stable long-term behavior, which in turn means more sensitivity to errors and more rapid adaptive responses.

The existence of a relationship between baseline activity and adaptation performance, especially if it can be further demonstrated in other motor processes, has important ramifications for any situation in which a treatment or intervention might be tailored to an individual’s adaptive capability. For example, the relationship between baseline variability and adaptation in saccades may be useful in the design of individualized rehabilitation protocols for patients with oculomotor disorders, particularly if a similar relationship can be demonstrated in other types of eye movements. To that end, in this current study we looked for an analogous result in the vestibulo-ocular system. Based on our results in the saccade system, we hypothesized that the strength of inter-trial correlations in a baseline VOR task, reflecting the use of prior performance information to modulate current and future behavior, would also relate to adaptability in the VOR.

## Materials and methods

### Experimental procedures

Twelve healthy individuals with no known vestibular, oculomotor, or neurological deficits volunteered as test subjects; two were later excluded due to excessive blinking during baseline tests. All subjects provided written, informed consent to a protocol approved by the Johns Hopkins Medicine Institutional Review Board. In this experiment, subjects performed a 7-min baseline VOR-gain test, followed by four 5-min blocks of VOR gain adaptation. During the baseline test and between the adaptation blocks, VOR gain was measured from simultaneous recordings of monocular, two-axis eye position (right eye, horizontal and vertical) and three-axis (roll, pitch, and yaw) head angular velocity (EyeSeeCam video-oculography (VOG), Munich Germany). Eye and head movement data were captured at 220 Hz and processed offline using algorithms described below.

The purpose of the 7-min baseline test was to provide a dataset from which fundamental properties of the VOR could be examined for comparison with adaptation performance. This baseline test was implemented as follows. Subjects viewed a stationary, centrally located point target 1.5 m away in an otherwise dark room. They repeatedly moved their heads rapidly in the yaw plane across the midline through an angle of approximately 30° (at approximately 300°/s peak velocity and 5000°/s^2^ peak acceleration), paced with a metronome at 60 beats per minute for seven minutes. This resulted in approximately 420 head-steps. One baseline VOR-gain value was derived for each head-step. Subjects were instructed to keep their eyes open for the duration of the test and only to blink, if necessary, between head movements.

Following the baseline test, subjects performed a 20-min VOR gain-adaptation paradigm, which consisted of four 5-min blocks of active, yaw-plane, sinusoidal head rotations while wearing x0.5 telescopic lenses. These lenses cause images to move half as fast as usual across the retinas during head motion, thereby requiring VOR gain to be reduced to 0.5 for proper oculomotor compensation. During the adaptation blocks, subjects focused on a stationary, centrally-located point target 1.5 m away and sinusoidally rotated their heads in yaw across the midline through an angle of approximately 40°; head movements were paced with a metronome set to 90 beats per minute, and subjects were instructed to complete one half-cycle per beat (0.75 Hz sinusoidal motion). Adaptation was performed in the light, and subjects were encouraged to simultaneously attend to their peripheral field of view to promote adaptation [16]. The active, continuous nature of the head movements in the light was intended to challenge the vestibulo-ocular system to promote gain adaptation as quickly and effectively as possible. Similar gaze-stability exercises are routinely given to vestibular hypofunction patients to facilitate compensation following unilateral or bilateral loss [17, 18]. Prior to the start of the adaptation protocol, and after each 5-min adaptation block, VOR gain was probed in complete darkness with a fixed, imaginary target, using 20 cycles (approximately 30 sec) of the same sinusoidal yaw head movements as during the adaptation blocks. (VOR gain was not measured during the adaptation blocks themselves.) The VOG system was removed before adaptation in order to don the minifying lenses, and vice versa. The head was stationary while donning and doffing the VOG system. However, in the course of the adaptation probes, a new method of assessing VOR gain was investigated in parallel with the main experiment on VOR adaptation. With the VOG system off, a set of head movements was made by the subject (25–50 head rotations, approximately one minute). During these movements, the subject nulled the perceived motion of a laser target at a distance of 1.5 m. Target motion reflected head motion, modified by a variable gain as set by the subject. Since the target was *not stationary in space* but rather was set to be *perceived as stationary* by the subject, this was not a de-adapting stimulus and should have had little or no effect on VOR gain. In fact our results, showing a monotonic decline in VOR gain across the adaptation procedure, support this contention. All subjects performed this procedure, which we again emphasize was not a de-adapting or washout stimulus.

Between adaptation blocks, subjects rested for 2 min with their eyes closed in complete darkness. At the beginning of the baseline and adaptation probes, a behavioral calibration was performed in which subjects fixated targets 8.5° up, down, left, and right of straight-ahead gaze. We define *adaptation extent* (Δ) as the difference in VOR gain between the probe just prior to the start of the first adaptation block and the last probe at the end of the 20-min adaptation period.

### VOR gain calculations

VOR gain is conventionally defined as the ratio of eye velocity to head velocity at the time of peak head velocity. However, differentiation (to obtain velocity from position) of the sinusoidal eye-position data obtained during the adaptation probes produced noisy eye-velocity traces, possibly subjecting peak detection to artifacts. Therefore, VOR gain values derived from the adaptation probes were instead computed from ratios of peak-to-peak eye position to peak-to-peak head position. Extraction of peak eye velocities from the 7-min baseline test, however, was easily achieved due to the high velocity and acceleration profiles, and so baseline VOR gain values for the head-steps were computed in the more conventional manner from the ratios of peak eye velocity to peak head velocity. (We did not perform a direct comparison of these two methods of finding VOR gain. Given the known relationship between position and velocity for sinusoidal motion, we expect that they are interchangeable. The replacement of saccades in the adaptation data was by interpolation over the removed segment, which produced a reasonable fit in position but introduced discontinuities that adversely affected the differentiation to velocity. Nevertheless, even in the absolute worst case that the two methods were measuring two different things, our overall results would remain valid, since we still would have meaningful and objective measures of variability and of adaptation. It is never the case that the adaptation measurements are mixed with the baseline measurements; only their summary statistics are compared.)

**Fig 1:**
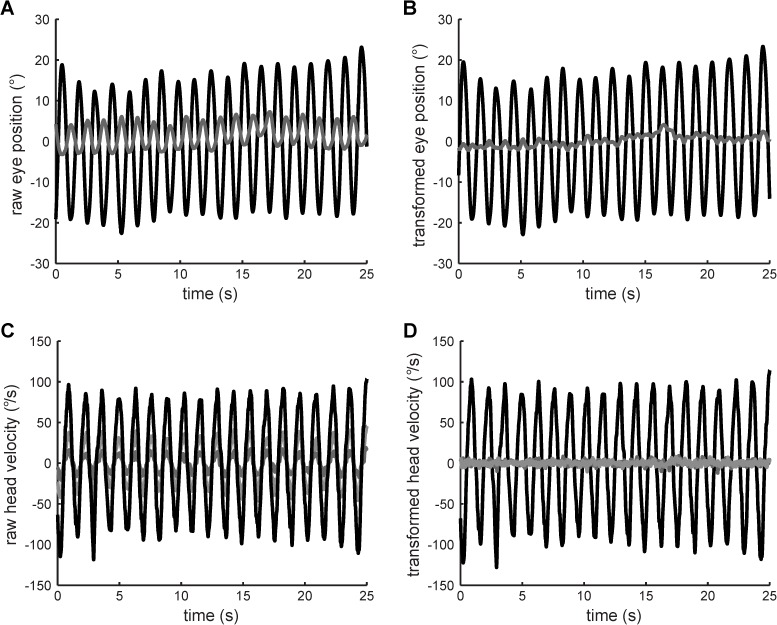
**Application of PCA to raw eye position and head velocity data of one VOR gain probe from one representative subject.** (A) raw eye data, (B) eye data transformed by PCA, (C) raw head data, (D) head data transformed by PCA. Principal components of the eye and head data are shaded black in (B) and (D).

Because the eye camera and the head-velocity sensor were oriented in different inertial reference frames, which were not necessarily in line with the body reference frame, principal component analysis (PCA) was applied to both the two-axis eye and three-axis head data (Fig 1). The resulting first principal components represent the yaw-plane eye and head motion in the body reference frame. All VOR gain computations were performed on these single-axis, first principal components.

For both the baseline and adaptation-probe datasets, fast phases and saccades within the eye-position data were automatically detected (and manually verified) based on a velocity threshold and were removed, and the resulting gap was filled with a linear regression estimate over the 25 ms before and after the fast phase. For the baseline datasets, peak eye and head positions were identified, and one VOR gain value was computed for each head-step; head-steps that contained blinks during peak head motion were eliminated. For the adaptation-probe datasets, linear trends were removed from the de-saccaded eye traces since testing was done in complete darkness to an imagined target, which occasionally allowed the subject’s perception of straight-ahead to drift [19]. Head velocity was integrated to obtain head position, and peaks in the eye and head position traces were detected. Half-cycles containing blinks were eliminated, and VOR gain values were computed for the remaining peak-to-peak pairs and then averaged to obtain a single gain value for each probe (five-minute interval).

### Quantifying inter-trial correlations

The relative strength of longer-term inter-trial correlations was estimated using spectral analysis of the baseline VOR gain data. Trial-to-trial fluctuations can be quantified with the autocorrelation function (ACF, denoted *R*_*xx*_(τ)). The ACF describes the linear cross-correlation between the signal and a time-shifted version of itself. For a stationary process *X*(t), the ACF is defined as

$$R_{xx}(\tau )=E\;\left[\frac{\left(X(t)-\mu )(X(t+\tau )-\mu \right)}{\sigma^{2}}\right]$$

where **E** is the expected-value operator, τ is the time shift between time series *X* and a copy of itself, and μ and σ^2^ are respectively the (sample) mean and variance of the time series *X*(t). For well-behaved processes (e.g., those for which the ACF is integrable), -1 < *R*_*xx*_(τ) < 1, with 1 indicating perfect correlation and -1 indicating perfect anti-correlation. The ACF is normalized such that *R*_*xx*_(τ = 0) = 1 indicating that a signal is perfectly correlated with an exact (non-time-shifted) copy of itself.

The decay of the ACF provides information regarding the strength and persistence of inter-trial correlations: how rapidly information from past trials is “forgotten.” For a completely uncorrelated white-noise process, *R*_*xx*_(τ≠0) = 0, meaning that there is no correlation among trials, as expected from a process without memory. Thus, a no-memory process has no inter-trial correlations. On the other hand, for integrated white noise, where each subsequent trial can be thought of as the sum of all previous trials, *R*_*xx*_(τ) = 1 for all τ, and the process has infinite memory, with uniformly strong inter-trial correlations. Between a no-memory process and an infinite-memory process is a continuum of different memory processes. Informally, we can consider processes as “longer-memory” when their ACF decay gradually, as indicative of longer-term correlations, and “shorter-memory” when their ACF decay more rapidly, as indicative of shorter-term correlations.

Longer-term correlations are reflected in low-frequency activity (coordinated action over long time spans), which is more easily seen in the frequency domain than in the long tails of the ACF. The power spectrum (or power spectral density, denoted *S*_*xx*_(*f*)) represents signal power in each frequency bin. It is the Fourier transform of the ACF. Longer-memory processes (i.e., those that exhibit stronger correlations over longer times) have ACFs that decay gradually and power spectra that contain a larger ratio of low-frequency relative to high-frequency power. We use the phrases “longer-term correlations” and “shorter-term correlations” to denote datasets that exhibit relatively larger or smaller proportions of low-frequency activity, respectively. The relative magnitude of low to high frequency power can be quantified by a line fit to the spectrum [20], with a (negative) slope β on log-log axes. β is thus a critical measure of the relative strength of longer-term to shorter-term inter-trial correlations, which we use extensively here. It is useful to think of β as quantifying the spectral “distance from white noise,” meaning the presence of stronger correlations. Larger β magnitudes represent more relative power in the lower-frequency portion of the power spectrum, and are thus indicative of stronger long-term correlations. Smaller (positive) β values represent more relative power in the higher-frequency portion of the power spectrum, and are thus indicative of stronger short-term correlations. (Negative β values are also indicative of shorter-term inter-trial correlations. They represent more relative power in the high-frequency portion of the power spectrum in comparison to a white-noise process, and are hence indicative of time series that tend to fluctuate about the mean more often than by chance.)

To minimize confusion and misinterpretation of our analyses, here we reserve the word *correlation* to describe inter-trial correlations. When referring to the mathematical linear relationship between two variables, we use phrases such as *linear trend*, *relation*, or *association*. Below, we define our primary finding of a linear trend as the *β–Δ result*.

### Verifying the reliability of inter-trial correlations and spectral estimates

There are several mathematical properties that a time signal must have in order to yield valid spectral estimates. Among these is stationarity, which implies that the statistics of the underlying distribution, including the mean and variance, do not change over time. Although a rigorous mathematical test of stationarity was not performed it is reasonably assumed for our data: subjects continually viewed a fixed target during the baseline head-steps, thereby centering the VOR gain around 1.0, and the total time of the baseline test was limited to seven minutes to minimize fatigue or boredom which might have altered the mean or variance during later trials.

In addition to stationarity, the time series should not contain substantial temporal gaps: large numbers of individual trials cannot be omitted as this would disrupt temporal correlations. Thus it is not appropriate to eliminate trials that, for example, appear to be outliers. Accordingly, VOR gain values for all baseline head-steps were included in the correlation analysis, with the exception of a small number of trials that contained blinks during the time of peak-head velocity (when VOR gain was measured) because no reliable eye-movement information was available in these cases. (Two subjects were removed for excessive blinking. The remaining ten subjects blinked in fewer than 3.6% of baseline trials. These trials were removed and all baseline datasets were truncated to 405 gain values.)

Finally, the time series should not contain outliers. Large-magnitude outliers represent randomly distributed impulse functions in the time domain, which lead to disruptive transients in the power spectrum that do not accurately represent the underlying data. Identification of outliers is described in the next section. (Outliers were not simply removed, as were blinks, due to their larger number and possible systematic nature.)

Given these concerns about the ability to obtain meaningful spectral estimates (and hence assessment of inter-trial correlations), we used the method of surrogate data [8, 21]. This allowed us to generate well-behaved data sets that reflect the critical defining features of the original data, but (by construction) are devoid of outliers and excess kurtosis. Spectra and β values were derived from these surrogates. The surrogates used here assume that the data can be modeled as a sequence of Gaussian random variables with the same rank order as the original data, in which the ordering of the relative magnitudes of the values is the important factor in determining the inter-trial temporal correlations. In other words, the rank order of the values is retained in each data set, while the values themselves are drawn from a Gaussian distribution.

All of the baseline datasets contained outliers, and so each subject’s baseline VOR gain values were replaced with rank-ordered Gaussian random variables (GRVs) so that the derived β values were not disproportionately biased by these random occurrences. The rank-ordered GRV surrogates were created in the following manner. For each subject, 405 Gaussian random variables were generated and then rank-ordered based on the amplitude rank-order of the raw baseline gain data. This process was repeated one hundred times to generate one hundred rank-ordered GRV surrogate datasets per subject. Mean β values were then calculated from these surrogates for each subject.

### Managing and assessing outliers in the baseline data

It is sometimes useful to examine the specific impact of outliers in physiological data, since they might be valid values as opposed to simply anomalies. Thus, we needed a way to systematically identify outliers in the baseline VOR data. Our criterion for designating outliers was those data points that precipitated excess kurtosis in the raw baseline data. We consider excess kurtosis to be greater than 3.0, which is the kurtosis observed in a Gaussian distribution. Outliers were delineated as follows. The mean and kurtosis of the original set of raw baseline gain values were computed. For all subjects, the kurtosis was larger than 3.0 due to the presence of the outliers. The data were then sorted based on their distance from the mean, and the data point furthest from the mean was removed. The mean and kurtosis were recomputed, and the kurtosis was once again checked against the value of 3.0. If the kurtosis was larger than 3.0, the data point farthest from the new mean was removed. This was repeated until enough outliers were eliminated, one at a time, to render the kurtosis less than 3.0. As a final verification, the skewness was computed to ensure that the underlying distribution was approximately symmetric. Those data points removed during the kurtosis-trimming procedure were designated as outliers.

There are two general theories regarding the nature of outliers. One is that they are simply noise: extraneous events that occur sporadically with no specific purpose. The other is that they are intentional, exploratory movements whose presence might modify subsequent behavior [9, 22]. If the outliers in our baseline gain data were exploratory, then one might expect the trial immediately following an outlier to be the most affected by that outlier. Therefore, we tested the null hypothesis (H_0_) that there is no difference in VOR gain between the trial just before and the trial just after an outlier. If our outliers were indeed exploratory, then we would expect to see systematic performance changes in the trial immediately following the outlier, as subjects adjusted their actions based on the outliers. However, if the outliers were simply random noise, then we would expect no difference in the gain values before and after an outlier occurred (i.e., no behavioral modifications as a result of the outliers). A linear random-intercept model incorporating separate intercepts for each subject was employed to test this hypothesis.

## Results

### VOR gain adaptation varied across subjects

**Fig 2:**
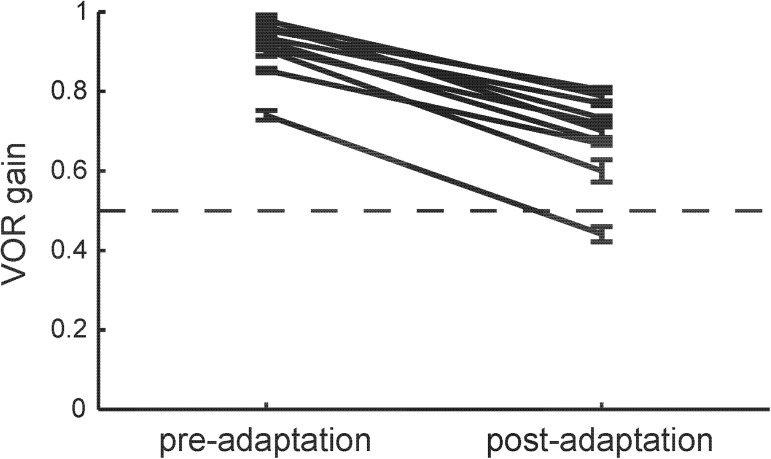
Pre- and post-adaptation VOR gain results.

The mean pre- and post-adaptation VOR gain values for each subject are shown in Fig 2. Recall that adaptation extent Δ was defined as the difference between these pre-adaptation and post-adaptation values. All subjects demonstrated significant changes in gain following the 20 min of adaptation (mean Δ ± s.d.: 0.225 ± 0.057), but the change varied across subjects. (VOR baseline values and mean adaptation values are available; see S1 File.)

### Baseline inter-trial correlations are strongly associated with adaptation performance

**Fig 3:**
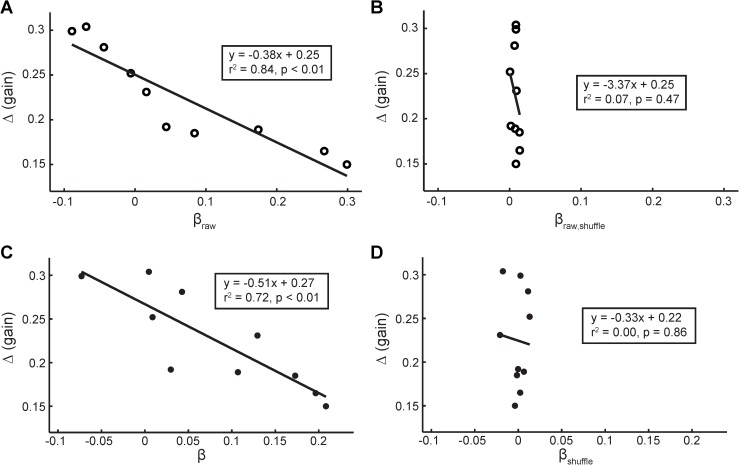
(A) Δ is strongly related to β_raw_ derived from the raw baseline VOR gain data. (B) Δ is not related to β_raw,shuffle_ derived from time-shuffled surrogates of the raw baseline VOR gain data. (C) VOR adaptation extent Δ is strongly related to β. (D) Δ is not related to β_shuffle_, derived from time-shuffled surrogates of the rank-ordered GRV surrogates.

VOR gain adaptation extent Δ exhibited a strong linear relationship with the β values derived from the baseline raw VOR data (β_raw_) (Fig 3A, r^2^ = 0.84, p < 0.01). We refer to this as the β-Δ result, and it is the main finding of our study. Specifically, the larger the β value (the stronger the longer-term, low-frequency correlations), the weaker the adaptive capacity (the less the VOR adaptation extent) of the individual. No relationship was found between spectral slope β and extent of adaptation at the intermediate gain probes (5, 10, or 15 min into adaptation).

To verify that the β-Δ result is not simply due to the statistical distribution of the baseline VOR gains but is indeed due to the temporal ordering of the values, the VOR gains were randomly shuffled to destroy temporal correlations. We then computed a new β_raw,shuffle_-Δ result using these time-shuffled surrogates. If inter-trial correlations are a significant contributor to the original β-Δ relationship depicted in Fig 3A, then randomly scrambling the temporal structure should result in β_raw,shuffle_ values close to zero (analogous to uncorrelated white noise) and no relation between β_raw,shuffle_ and Δ. This is in fact what happens (Fig 3B).

### Baseline VOR gain data contain outliers, but this does not alter the β-Δ relationship

All subjects demonstrated baseline VOR-gain values with means approximately equal to 1.0, but with variability from trial to trial. Between 8 and 40 outliers (approximately 2–10% of the total number of baseline trials) were present in our ten subjects’ baseline datasets, which is consistent with the literature regarding repeated VOR-gain measures in healthy individuals [23, 24].

The existence of the β-Δ relationship in the presence of VOR-gain outliers leads to the question of whether the outliers–given their magnitudes–contribute disproportionately to the inter-trial correlations or to the relationship between β and adaptation rate. This was tested through a data transformation in which the outliers maintain their original temporal locations, while the non-outliers are randomly shuffled in time. If the outliers are the predominant factor, then the overall β-Δ relationship should be maintained, even as the temporal correlations within the majority of the data (the non-outliers) are disrupted. No such relationship was observed when these transformed datasets were generated from either the raw data or the rank-ordered GRV surrogates described below (r^2^ < 0.15, p > 0.3), and hence, it is highly unlikely that our result is due to a dominating effect of the outliers.

### Outliers are not related to systematic changes in VOR performance

We consider outliers to be anomalous values in our analyses, but it is possible that they serve a role in exploratory behavior that might aid adaptation. To test the potential for a systematic role in motor learning due to the outliers, a linear random-intercept model was fit to estimate the difference in the mean pre-outlier versus post-outlier VOR gain values; the random intercept was included to account for the correlation in observed gain values within a subject. The model revealed no significant difference in pre-outlier versus post-outlier gain values (χ^2^(1) = 0.79, *p* = 0.43). It was therefore concluded that the outliers were simply random, sporadic occurrences that did not systematically alter behavior.

### Surrogate data analysis to address outliers verifies the β-Δ result

**Fig 4:**
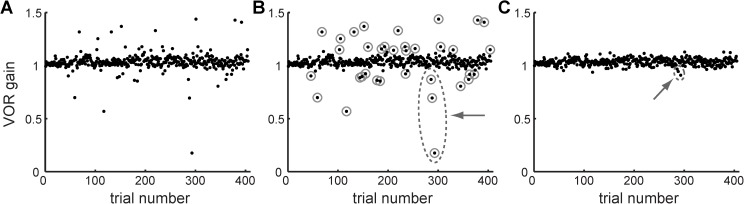
**Baseline VOR gain data from one representative subject.** (A) raw data, (B) raw data with outliers circled, (C) sample rank-ordered GRV surrogate data. Grey dashed circles and arrows demonstrate how the rank-ordered amplitude is preserved in the surrogate.

**Fig 5:**
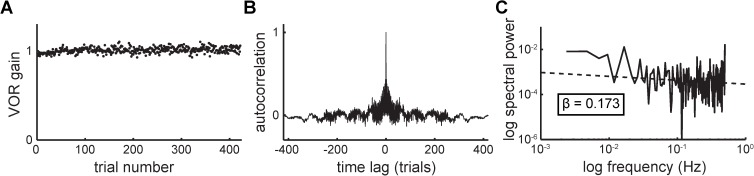
**Data from one representative subject.** (A) rank-ordered GRV surrogate dataset, (B) corresponding autocorrelation function, and (C) corresponding power spectrum.

Due to the presence of outliers that might impact the spectral estimates, the raw baseline data were replaced with rank-ordered GRV surrogates (see Methods). The raw baseline data from one subject are displayed in Fig 4A with the outliers circled in Fig 4B. One rank-ordered GRV surrogate for this individual is displayed in Fig 4C. Note that while the rank-ordered GRV transformation effectively “reigns in” the outliers, their outlier “status” has not been changed: the trials furthest from the mean in the original raw data are the same trials that are furthest from the mean in the surrogate data (Fig 4B and 4C dashed ellipses and arrows). The ACF and power spectrum were computed for each subject’s baseline surrogate datasets, as for the raw values described above. A sample rank-ordered GRV surrogate dataset and its corresponding ACF and power spectrum are displayed in Fig 5.

The relationship between VOR adaptation extent Δ and β_raw_ described previously (β derived from the raw baseline VOR gain data) is also present using the rank-ordered GRV surrogates (Fig 3C, r^2^ = 0.72, p < 0.01). This verifies that statistical anomalies in the VOR data (nonstationarity, outliers) do not have an adverse effect on the spectral estimates, and establishes that rank-ordered GRVs constitute a reasonable model for these data. It is the temporal ordering of the relative ranks that establishes the inter-trial correlations that are then reflected in β.

Furthermore, no relationship exists between VOR adaptation data and β_shuffle_ (β derived from time-shuffled versions of the GRV surrogate data), as all β_shuffle_ values collapse to zero (Fig 3D, r^2^ = 0.00, p = 0.86).

**Fig 6:**
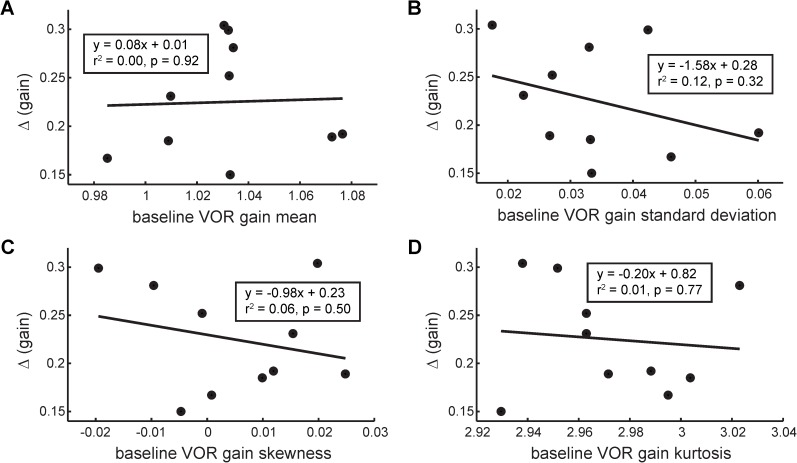
No correlation is observed between VOR adaptation extent and the mean (A), standard deviation (B), skewness (C), or kurtosis (D) of the baseline rank-ordered GRV surrogates.

The main finding of a relationship between β and Δ suggests that trial-to-trial fluctuations in baseline VOR gain data may reflect the ability of the VOR to adapt to novel perturbations. Since others have posited that simpler measures of variability may be related to adaptive capabilities [11], we also examined whether standard statistical parameters of the baseline VOR-gain data–mean, standard deviation, skewness, kurtosis–could be linked to adaptability. No such relationship was found (Fig 6, r^2^ < 0.12, p > 0.32). This validates our hypothesis that it is not variability *per se*, but rather fluctuations in the trial-to-trial time ordering of the successive gain values (i.e., the temporal structure), that reflects neural processing of performance that is related to adaptation.

## Discussion

The strong β-Δ relationship that we find here suggests that the relative strength of longer-term to shorter-term inter-trial correlations in consecutive baseline VOR gain values (i.e., the temporal structure, quantified by spectral slope β) can provide a foundation for forecasting the adaptive capacity of the VOR on an individual basis. This raises the intriguing possibility of being able to forecast an individual’s adaptive capability in a given task, based on a (possibly fractal) measure of that person’s ability to store and process information on sensorimotor errors in a related task. Given the importance of the VOR in everyday activities, and the emphasis placed on physical therapy and rehabilitation to recover VOR function in vestibular patients, the ability to forecast adaptation ability could have significant and far-reaching implications. As another example, in human space flight it would be highly advantageous to know ahead of time which crewmembers might have more difficulty adjusting to the novel g-levels inherent to spaceflight. This type of knowledge could guide individually customized countermeasures to maximize crew safety and mission success.

### Temporal fluctuations relate to adaptive capabilities in the VOR

Trial-to-trial variations in motor performance are typically interpreted as irrelevant and bothersome “noise.” Our results instead suggest that such temporal fluctuations may reflect an active, intentional mechanism that can be related to adaptive capabilities. We find that the strength of inter-trial correlations in a sequence of VOR gain values is related to the ability to adapt to a visual-vestibular perturbation that calls for a change in VOR gain. Subjects whose baseline VOR-gain data exhibit relatively longer memory (i.e., power spectra with larger ratios of lower-frequency to higher-frequency content) are those who adapt the least, while subjects who demonstrate shorter memory (i.e., power spectra with relatively smaller ratios of lower-frequency to higher-frequency content) adapt the most. In fact, the β values (spectral slopes) associated with subjects who adapt most are negative, which indicates that during baseline testing, VOR performance with each head movement primarily depends on what occurred only in the most recent trial. Notably, the parameter associated with adaptation (β) depends specifically on the temporal order of the individual trials.

Here, the extent of VOR adaptation was derived from pre- and post-adaptation (after 20 min) probes of VOR gain. There was no relationship between spectral slope β and adaptation extent derived from earlier gain probes (5, 10, or 15 min into the adaptation). This is most likely because adaptation had not reached a stable state until 20 min, and hence the probe at 20 min is a more accurate reflection of adaptation capability across all individuals despite differences in adaptation rate. Moreover, since the VOR gain could only be assayed at a few discrete time points during learning, fits of adaptation rate are likely to be quite noisy and therefore may not provide a good representation of adaptation capacity, in contrast to our prior work examining saccade adaptation [15] in which rate was a more robust measure of adaptation capacity.

### VOR baseline data do not exhibit fractal structure

Within broad constraints, if the ACF decays as a power law, then the decay of the power spectrum also follows a power law [25], producing a straight line on a log-log frequency plot. Such power-law behavior is indicative of a fractal time series, in which there is (statistical) self-similarity across different time (frequency) scales. Based on the results of several fractal-analysis techniques we do not have convincing evidence that our baseline VOR data represent such a fractal process, while our previous saccade data are fractal [26]. Nevertheless, the mathematical tools and concepts used to assess fractal behavior, and especially the relative magnitudes of low- and high-frequency activity (longer- and shorter-term correlations, respectively), still provide useful insights [20].

The essential point remains, moreover, that even though the correlations might not be self-similar (fractal), any temporal correlations introduce structure in the spectrum and distinguish it from that of a white-noise process with a flat spectrum (slope of zero, lack of inter-trial correlations). Therefore, β continues to provide an estimate of this relationship between longer-term and shorter-term inter-trial correlations.

### The VOR and saccadic systems both seek to maximize time of useful vision

The relationship between β and adaptation capacity observed here is in direct contrast to our previous findings in the saccadic system [15], where larger β in a baseline saccade task corresponds to better adaptation ability. However, this seeming discrepancy might be resolved by considering the ultimate goal of both oculomotor systems: to maintain stable images on the retina using whatever adaptive adjustments are most readily available to each system. This is particularly evident in the VOR, which works to minimize slip of images on the retina. Indeed, the presence of rapid online corrections during active head motion in the presence of continuous visual feedback suggests that this system is particularly concerned with ensuring that the VOR gain at every instant is as accurate as possible, to avoid the need to introduce catch-up saccades (maximizing the time that vision is available). To that end, the VOR should immediately respond to any inaccuracies in gain, resulting in a system that is highly concerned with the most recent error and much less concerned with any errors from the past. That is, the ideal VOR system would adapt as much as possible on every trial in response to the immediate error experienced on that trial in order to keep the gain close to 1.0. This could be considered *within-trial* learning as opposed to *trial-to-trial* learning. Better adaptation capability would then be reflected in smaller β values (less storage from trial to trial) at baseline. For the same reason (to minimize per-saccadic degradation of vision) the saccadic system exhibits the opposite behavior. It is more costly to make a large saccade that overshoots the target which then requires a backward correction, than to make a shorter saccade and a small forward correction [27, 28]. Hence, the saccadic system favors maintaining a stable hypometric gain despite fluctuations from trial to trial. In order to determine the appropriate saccade gain, then, this system should rely on performance information from a large number of trials in the past (yielding a larger β). Because the saccadic system has so much information at its disposal, this also could make it sensitive to large unexpected errors such as those introduced in an adaptation paradigm. Therefore, we would expect (and in fact observe) that subjects with larger β values at baseline can detect and respond to errors more rapidly and display greater adaptation capability. The same underlying goal of maximizing time of stable vision thus can account for the opposite relationships between β and adaptation capability that we observe here and in our previous work in saccades.

### Study limitations

One limitation of this study is the use of slightly different measures for VOR baseline assessment (step-like head movements) and for VOR adaptation assessment (sine-like head movements). Partly this selection was made in order to obtain high-quality data in each condition while accommodating the ease and comfort of the test subjects. Rapid impulsive head movements provide a clear, strong vestibular stimulus, allowing for straightforward delineation of individual trials and thus making gain assessment simple and reliable. Lower-frequency sine-like movements are easier for subjects to maintain with the consistency required for an adaptation experiment, and they reflect part of the range of natural active movements made in normal life. The most important consideration, however, is that we desired a set of baseline (predictor) gain values that were clearly discrete, so as to mimic the saccade data in our companion experiment [15]. This yields a more meaningful measure of temporal (inter-trial) correlations than would be the case if the movements were continuous and sequential trials not clearly delineated.

### Functional implications and interpretations

In light of the current VOR results and the previous work on saccade adaptation, one might posit that an individual’s propensity to retain motor-performance information might be a global characteristic of that individual’s sensorimotor processing (a “sensorimotor phenotype”). In other words, if an individual exhibits strong inter-trial correlations in one sensorimotor system, is it the case that he or she also expresses strong inter-trial correlations in other systems? Such a finding would mean that characterizing baseline performance in one system would not only lead to predictions regarding adaptive capacities in that system, but also to predictions about adaptive performance in other systems. To this end, we compared the β values and adaptation performance across six subjects who participated in both the saccade [15] and VOR (this study) experiments. There was no systematic relationship between β and adaptation performance across the two experiments. The β values derived from the baseline saccade data were not the same β values derived from the baseline VOR gain data, and those subjects who were high-performing adapters in one experiment were not necessarily low-performing adapters in the other experiment.

However, this does not rule out the possibility that β may be a global sensorimotor predictor across systems that have similar requirements to store and process information on previous performance. The parameter may have an important predictive role in posture or locomotion, for example. Since many parameters of the locomotor system can be modified during motion (e.g., stride length and timing), perhaps the β values derived from VOR gain data would correlate with some aspect of performance during walking. Future experiments are needed to assess such cross-system possibilities. Additionally, even if measures of β do not generalize at all across motor systems, the ability to find a unique relationship between β and adaptation capacity for any given motor system still provides important insight into the nature of what parameters that system is seeking to optimize.

Accuracy of sensorimotor behaviors is consistently maintained through learning and adaptation. This has been convincingly demonstrated for a great many behaviors, including saccades, reaching, locomotion, and vestibular reflexes. A major component of these adaptive processes is error-based learning: the errors of previous movements are monitored and used to alter subsequent movements in order to make them more accurate. An important feature of these types of motor learning is that error is stored and appropriately processed. Such trial-to-trial learning can be modeled as a Markov process when the single previous trial has the dominant effect, or more generally as state-space models with various memory times. Our results here show that these memory times and model orders are critical parameters that can connect baseline performance and adaptive ability.

## Supporting information

S1 File Supplementary information file “VOR gains supplement.xls” contains data on the extent of VOR adaptation, and the raw VOR gain values from baseline testing. (XLS)

## Abbreviations

ACF: autocorrelation function
g-level: gravity level
GRV: Gaussian random variable
PCA: principal component analysis
VOG: video-oculography
VOR: vestibulo-ocular reflex
B: spectral slope: relative strength of longer- and shorter-term inter-trial correlations
Δ: adaptation extent: difference between pre- and post-adaptation VOR gains
